# Anti-Virulence Therapeutic Approaches for *Neisseria gonorrhoeae*

**DOI:** 10.3390/antibiotics10020103

**Published:** 2021-01-21

**Authors:** Katherine Y. L. Lim, Christopher A. Mullally, Ethan C. Haese, Emily A. Kibble, Nicolie R. McCluskey, Edward C. Mikucki, Van C. Thai, Keith A. Stubbs, Mitali Sarkar-Tyson, Charlene M. Kahler

**Affiliations:** 1 Marshall Centre for Infectious Disease Research and Training, School of Biomedical Sciences, University of Western Australia, Crawley, WA 6009, Australia; katherine.lim@research.uwa.edu.au (K.Y.L.L.); christopher.mullally@research.uwa.edu.au (C.A.M.); ethan.haese@research.uwa.edu.au (E.C.H.); emilyalice.kibble@murdoch.edu.au (E.A.K.); nicolie.mccluskey@murdoch.edu.au (N.R.M.); edward.mikucki@research.uwa.edu.au (E.C.M.); vanchi.thai@research.uwa.edu.au (V.C.T.); mitali.sarkar-tyson@uwa.edu.au (M.S.-T.); 2 School of Veterinary and Life Sciences, Murdoch University, Murdoch, WA 6150, Australia; 3 School of Molecular Sciences, University of Western Australia, Crawley, WA 6009, Australia; keith.stubbs@uwa.edu.au

**Keywords:** *Neisseria gonorrhoeae*, antimicrobial resistance, sexually transmitted infections, virulence factors, anti-virulence therapy

## Abstract

While antimicrobial resistance (AMR) is seen in both *Neisseria gonorrhoeae* and *Neisseria meningitidis*, the former has become resistant to commonly available over-the-counter antibiotic treatments. It is imperative then to develop new therapies that combat current AMR isolates whilst also circumventing the pathways leading to the development of AMR. This review highlights the growing research interest in developing anti-virulence therapies (AVTs) which are directed towards inhibiting virulence factors to prevent infection. By targeting virulence factors that are not essential for gonococcal survival, it is hypothesized that this will impart a smaller selective pressure for the emergence of resistance in the pathogen and in the microbiome, thus avoiding AMR development to the anti-infective. This review summates the current basis of numerous anti-virulence strategies being explored for *N. gonorrhoeae*.

## 1. Introduction

*Neisseria gonorrhoeae* is a Gram-negative diplococcus which causes the sexually transmitted infection (STI) gonorrhea. The World Health Organization (WHO) estimates that of the 376 million new cases per annum of treatable STIs (chlamydia, gonorrhea, syphilis and trichomoniasis), *N. gonorrhoeae* caused 87 million cases globally [1]. Specifically, in the United States, gonorrhea is the second most commonly reported notifiable infection. A 2018 surveillance report by the Centers for Disease Control and Prevention determined that a total of 583,405 cases had been recorded, an 82.6% increase from the historic low observed in 2009 [2]. A study on the total lifetime direct medical cost of gonorrhea infections on the US healthcare system was approximately $81.1 to $243.2 million [3]. However, this cost does not reflect the true economic burden of *N. gonorrhoeae* infections since it did not include costs associated with adverse pregnancy outcomes, disease prevention or productivity loss.

*N. gonorrhoeae* most commonly colonizes the genital mucosa, but can also colonize the ocular, nasopharyngeal and anal mucosa. Gonococcal infections in men are predominantly symptomatic, but pharyngeal and rectal infections in men are overwhelmingly asymptomatic. Symptomatic patients usually present with acute urethritis, displaying symptoms of dysuria and urethral discharge [4,5,6,7,8]. On the other hand, infections in women are frequently asymptomatic, with some studies indicating up to 70% asymptomatic infection rates [9]. Symptomatic infections of the genital mucosa usually manifest as cervicitis, urethritis and occasionally as pelvic inflammatory disease (PID) [10]. Asymptomatic cases are reservoirs that promote gonorrhea transmission, and undetected AMR strains from these reservoir sites may promote the spread of resistance.

Gonococcal urethritis significantly increases the risk of acquiring and transmitting HIV, thus substantially contributing to the public health burden of this infection [11,12,13,14,15]. Genital infections in pregnant women can have adverse effects on the fetus including spontaneous preterm birth, chorioamnionitis, low birth weight, premature rupture of membranes and spontaneous abortion [16,17]. Additionally, transmission to the neonate may occur during passage through the birth canal. The effects of gonococcal disease for neonates include severe eye infections and bacteremia that can lead to ulceration of the cornea, perforation of the globe of the eyes or permanent blindness [18,19,20,21].

To date, no successful vaccine strategies have been developed for gonorrhea in humans, as individuals can contract the disease multiple times throughout their lifetime, suggesting that there is no natural immunity and therefore correlates of protection to benchmark vaccine efficacy [22,23]. Recent studies have observed an association of reduced prevalence of gonorrhea in individuals who have received the *N. meningitidis* serogroup B vaccine Bexsero^®^, suggesting that there may be cross-protective immunological responses elicited from common antigens in the meningococcal outer membrane (OM) vesicle component [24,25]. Further work is required to fully analyze the immune response elicited by this vaccine, but this provides a framework for future gonococcal vaccines, and reinforces the requirement for human clinical trials to identify successful vaccine antigens [26].

## 2. Treatment and Antimicrobial Resistance

All gonococcal infections are treated with antibiotics, but different regimes may be recommended depending on the site of infection. For urethral, anorectal and oropharyngeal infections, the WHO recommends a dual therapy of 250 mg of intramuscular ceftriaxone as a single dose and 1 g of oral azithromycin as a single dose [27]. Alternatively, 400 mg of oral cefixime can be administered as a single dose in conjunction with a single 1 g dose of oral azithromycin. The dual therapy treatment for gonococcal infections is designed to prevent the ever-increasing levels of antibiotic resistance observed in *N. gonorrhoeae*. Neonatal gonococcal conjunctivitis should be treated with 50 mg/kg intramuscular ceftriaxone as a single dose, 25 mg/kg intramuscular kanamycin as a single dose or 25 mg/kg intramuscular spectinomycin as a single dose [27]. Ocular prophylaxis after birth should also be applied to infants following perinatal cervical exposure using topical treatments such as tetracycline hydrochloride or erythromycin eye ointment [28].

It is of great concern that AMR has risen to the point where there now exists no known class of antibiotics to which resistance has not been identified [29,30,31,32,33,34,35,36,37,38,39,40]. The cost of healthcare treatment for AMR infections is higher than for common infections since patients often have extended hospital stays, and require more intensive and expensive care [41,42,43,44,45].

Development of antibiotics against AMR *N. gonorrhoeae* has been underway for some time and many clinical candidates such as solithromycin, zoliflodacin, SMT-571 and gepotidacin have entered clinical evaluation for treating uncomplicated gonorrhea [46,47,48,49,50]. Unfortunately, mechanisms for resistance against these antimicrobials are already present in the bacterial population as the targets chosen are not novel. In addition, the suitability of some of these compounds to treat gonorrhea has been reduced due to pharmacological issues such as longevity and stability in the urogenital compartment. There also reports of higher rates of oropharyngeal antibiotic treatment failures compared to other infection sites which have been attributed to the inability of the antibiotic(s) to reach a sufficiently high concentration in the oropharyngeal region [51,52]. Several studies by Chow et al. [53,54,55] that looked into the effectiveness of antibacterial mouthwash in treating oropharyngeal gonorrhea among men who have sex with men were unsuccessful or halted early due to high treatment failure rates, indicating the potential hurdle which oral treatments will have to overcome to reach gonococci present in the oropharynx.

## 3. Pathogenesis Mechanisms of *N. gonorrhoeae*

Following transmission from an infected to uninfected host, the gonococcus adheres to the apical side of the epithelial cells. This is mediated through gonococcal surface structures such as type IV pili (tfp), opacity (Opa) proteins, lipooligosaccharide (LOS) and the major OM protein porin, PorB [56]. Tfp, LOS and Opa can undergo both phase and antigenic variation during infection that minimizes recognition and elimination by the immune system [57].

Primary attachment is initiated by tfp which bind to the host cell surface receptor CD46 and/or complement receptor 3 [58,59]. In vitro studies indicate that antigenic variation of tfp influences pilus-mediated adherence to human tissue, colony morphology and DNA transformation efficiency [60,61]. To promote further intimate attachment, Opa proteins, which are phase variable [62], adhere to the carcinoembryonic antigen-related cell adhesion molecule (CEACAM) receptors, but some variants can bind to heparan sulfate proteoglycans (HSPGs) on host cells [58,63,64,65,66]. Attachment is also mediated by gonococcal LOS, which binds specifically to the host asialoglycoprotein receptor on HepG2 cells [67], human sperm cells [68] and epithelial cells [69]. Following adhesion, *N. gonorrhoeae* replicates to form microcolonies and biofilms [70,71], and some bacteria can proceed to invade epithelial cells by transcytosis [72,73,74]. During infection, gonococci releases fragments of bacterial LOS, peptidoglycan (PG) and OM vesicles during cell growth that activate two pattern recognition receptors, toll-like receptor (TLR) and nucleotide-binding oligomerization domain-like receptor (NOD) on epithelial cells, macrophages and dendritic cells [75,76,77,78,79]. *N. gonorrhoeae* also releases heptose-1,7-bisphosphate, a precursor for the incorporation of heptose into LOS, which activates TNF receptor-associated factor-interacting protein with forkhead-associated protein A (TIFA)-dependent immunity [80,81]. Activation of these TIFA, NOD and TLR signaling pathways leads to the activation of inflammatory transcription factors and release of pro-inflammatory cytokines and chemokines (e.g., IL-6, IL-8, CXCL3, CXCL10 and TNF-α) [58,82,83]. In response to these signals, large amounts of polymorphonuclear leukocytes (PMNs) are recruited to the site of infection, where *N. gonorrhoeae* is recognized and phagocytosed. Since gonococci can survive and replicate within PMNs, the massive influx of PMNs forms an observable purulent exudate that facilitates transmission [84].

## 4. Resistance of Gonococcus to Killing by Macrophages and PMNs

*N. gonorrhoeae* can avoid clearance by the immune system through a variety of mechanisms, including manipulating phagocytosis, modulation of the oxidative burst, defending against toxic neutrophil products and extending the neutrophil lifespan. Macrophages and PMNs are both phagocytic cells which utilize oxidative and non-oxidative mechanisms in microbial killing and degradation [85,86].

*N. gonorrhoeae* has four major mechanisms through which it is resistant to reactive oxygen species (ROS): quenching ROS, detoxification of ROS, maintaining redox homeostasis, and repair of oxidative damage. ROS can be quenched through a manganese (Mn) uptake system that uses Mn(II), encoded by the gene locus *mntABC* [87,88,89]. Detoxification of ROS occurs primarily through the expression of a cytoplasmic catalase, *katA* [90]. Additionally, *N. gonorrhoeae* can also maintain redox homeostasis through the production of glutathione, encoded by *gor* [91], while superoxide resistance is mediated by the periplasmic antioxidant Sco [92]. Finally, *N. gonorrhoeae* can protect nucleic acids from ROS through recombination repair mechanisms. It has been shown that several enzymes, including RecA, members of the Ref-like and RecBCD pathways, and Holliday junction resolvases RuvAC and RecF, all contribute to gonococcal survival after exposure to ROS [93]. Other enzymes such as RecN, PriA (replication restart enzyme), UvrABCD (nucleotide excision repair system), and MsrA/B have all been implicated in repair of oxidative damage [94,95].

Multiple mechanisms are employed by the gonococcus to evade the non-oxidative killing mechanisms of macrophages and PMNs. Phosphoethanolamine (pEtN) modification and sialylation of LOS results in increased resistance of bacteria to antimicrobial components, such as cationic antimicrobial peptide (CAMP) LL-37, while pili and porins have been reported to inhibit the release of antimicrobial substances [96]. Zughaier et al. (2015) [83] showed that pEtN modification of the lipid A moiety of the LOS reduced autophagy pathways in RAW 264.7 murine and human THP-1 macrophages. Additionally, the modulation of cellular iron metabolism has been reported to facilitate the survival of bacteria inside macrophages [97]. Gonococci have also been known to suppress immunity by polarizing macrophages and upregulating inflammatory and immunosuppressive cytokines (IL-6 and IL-10, respectively) [98]. The efflux pump systems have been shown to protect bacteria against the killing mechanisms of immune cells. The Mtr (multiple transferrable resistance) efflux pump system, MtrCDE, plays an important role in enhancing gonococcal survival during vaginal tract infection in mice models [99]. This efflux system also contributes towards extracellular survival, PMN extracellular traps and to PMN-derived antimicrobial peptides [100]. FarAB, another efflux system, also exports host-derived antimicrobials, but the exact mechanism of how this system contributes to bacterial defense against immune cells remains unknown [99].

## 5. AVTs as an Intervention Strategy

**Figure 1:**
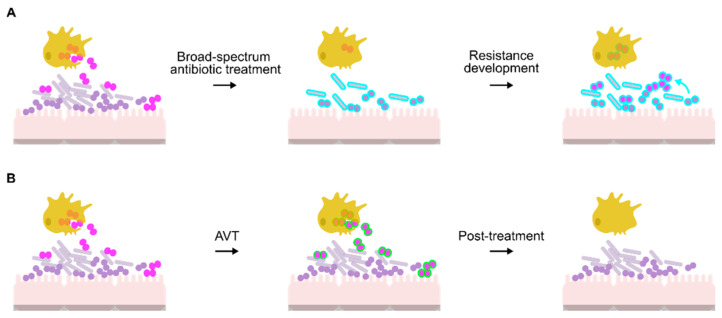
Comparison of antibiotic and anti-virulence approaches for treatment of *N. gonorrhoeae*. (**A**) Potential route for antibiotic resistance development in *N. gonorrhoeae* (pink diplococci). Antibiotics also affect the microbiome (*Lactobacilli*: grey rods; commensal *Neisseria*: purple circles), forcing resistance determinants to evolve (blue outline). This resistance can then develop in *N. gonorrhoeae* through gene acquisition via natural transformation (blue arrow) or spontaneous mutation. While antibiotic treatment may assist PMNs and macrophages (yellow cells) in killing the bacteria, the prescribed concentration may not be effective, resulting in proliferation of antibiotic resistant bacteria. (**B**) Use of AVTs (green outline) enables PMNs and macrophages to kill the gonococci without affecting the microbiome.

Antibiotic resistance in bacteria is driven by exposure to antibiotics. This exposure can occur via the food chain which delivers subtherapeutic concentrations of drug in the diet that drive the development of resistance in the microbiome. During antibiotic treatment of acute symptomatic infections [101,102], the majority of the human microbiome is removed, leaving resistant strains to proliferate and donate genetic markers of resistance via horizontal transfer mechanisms to colonizing pathogens (Figure 1A). In the case of *N. gonorrhoeae*, resistance markers evolve in the commensal *Neisseria* species of the human microbiome or in response to repeated antibiotic treatment failures. As this genus is naturally transformable, the pathogenic gonococci acquire the genetic markers via transformation and homologous recombination, in addition to in situ evolution of mutations in antibiotic target genes [103,104,105].

Anti-virulence therapies (AVTs) are compounds that target virulence pathways required for microbial pathogenesis in the host but are not essential to the growth of the pathogen in standard laboratory conditions [106]. Tailoring the AVT towards targets unique to the pathogen reduces selective pressure on the commensal flora, which remains intact and therefore is unable to become a reservoir of resistance determinants (Figure 1B). In the specific case of *N. gonorrhoeae*, preservation of the vaginal microbiome could also protect against gonococcal re-infection [107].

## 6. Gonococcal Virulence Factors as Targets for Inhibitor Design

**Figure 2:**
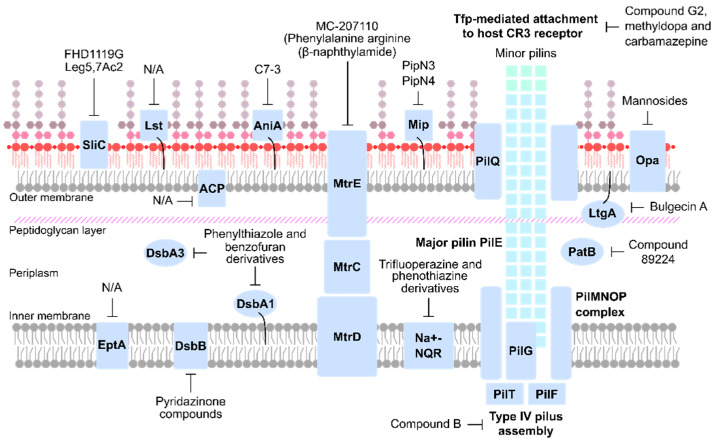
Overview of anti-virulence therapeutic targets of *N. gonorrhoeae*.

An ideal anti-virulence target should be found in all disease-causing strains and be essential for pathophysiology. Multiple compartments within the bacterial cell, including the cell wall, OM and secreted components fit these criteria (Table 1) and are summarized in Figure 2. Known anti-virulence targets and drug discovery programs against gonococcal virulence factors are explained in detail in the following sections.

### 6.1. Bacterial Cell Wall Maintenance and Modification

The gonococcal cell wall is characterized by the presence of both an inner membrane (IM) and OM separated by a PG layer. The PG is made up of linear glycan strands (repeating units of alternating *N*-acetylmuramic acid and *N*-acetylglucosamine residues joined through β-1,4 glycosidic bonds) cross-linked by short peptides [194,195,196]. The outer leaflet of the neisserial OM is composed of LOS, which consists of a membrane-anchoring lipid A domain and an inner core of 3-deoxy-D-manno-2-octulosonic acid linking it to a polysaccharide core [197]. Lipid A comprises a di-glucosamine backbone, 1- and 4’-phosphate groups and six acyl chains [198,199]. Since the PG and OM provide a substantial protective barrier, targeting enzymes that preserve or remodel the PG, such as acetylases and lysozyme inhibitors [121,122,124,200], and LOS components could represent promising novel drug targets for treating MDR gonococcal infections.

#### 6.1.1. Lipid A Phosphoethanolamine Transferase

The modification of lipid A with pEtN is mediated by the enzyme lipid A phosphoethanolamine transferase (EptA). EptA adds pEtN to the 1 and/or 4′ positions of lipid A [108,201] and is a characteristic virulence factor of pathogenic *Neisseria* [202] that affects multiple aspects of gonococcal survival. The presence of the positively charged pEtN affects the neisserial cell surface and gonococcal strains lacking pEtN modification have been proven to be more susceptible to CAMPs and complement-mediated killing [109,203,204]. In addition, further studies have shown that *eptA* knockout strains are highly susceptible to killing by human PMNs and macrophages [83,111]. Loss of pEtN decoration was also found to decrease binding of LOS by the host TLR-4/MD-2 signaling pathway and lower cytokine expression [77,202]. EptA is also essential for survival in the murine female genital tract and in human male volunteers. A study by Hobbs et al. (2013) [110] showed that in competitive inhibition assays in mice, there was a minimum of 10–10,000-fold reduction in *eptA* mutant strain recovery compared to the wild type. No *eptA* mutant strains could be recovered after day 6 post-inoculation in mice, and in human volunteers, *eptA* mutants could not be recovered at any point in time post-inoculation.

The enzyme EptA is a particularly attractive target as it is essential for pathogenesis, is the only lipid A-modifying enzyme present and is found in all strains of pathogenic *Neisseria*. While there are no studies currently published on the development of inhibitors targeting EptA, this enzyme is a promising target for structure-based drug design. Crystallographic and functional studies have highlighted residues in the catalytic site where inhibitors can be designed to target, thereby reversing the resistance of the gonococci to antimicrobial peptides [112,201,205,206].

#### 6.1.2. LOS Sialyltransferase

The α-chain of LOS is variable due to the differing expression of LOS glycosyltransferases (Lgt), which sequentially add glycan residues to the α-chain extending from HepI [207]. The expression of certain α-chain structures which mimic host glycans, such as lacto-*N*-neotetraose (LNT), play an important role in attachment to and invasion of the host epithelium and immune invasion in both gonococci and *N. meningitidis* [63,208]. In particular, the sialylation of gonococcal LNT with sialic acid (Neu5Ac) by LOS sialyltransferase (Lst) has been shown to confer serum resistance when grown in media supplemented with cytidine monophospho-*N*-acetylneuraminic acid (CMP-NANA, the donor molecule for Neu5Ac) [209,210,211,212,213] by interfering with all three complement activation pathways [213,214,215,216]. The *lst* gene is ubiquitous among gonococcal isolates [217], and is actively expressed following contact with host cells under the control of the transcriptional regulator CrgA [218], making it an attractive potential target for anti-virulence therapies [219].

Two major anti-virulence strategies targeting LOS sialylation have been investigated to date. One strategy made use of chimeric proteins consisting of factor H linked to the Fc domain of murine IgG—termed FH/Fc [117]. By mimicking factor H mutations observed in atypical hemolytic uremic syndrome (a condition resulting in the overactivation of the alternative complement pathway), Shaughnessy and colleagues created a variant of FH/Fc, FHD1119G, which was non-toxic to host cells but could bind to multiple sialylated clinical isolates of *N. gonorrhoeae*, including ceftriaxone-resistant isolates, to varying degrees. FHD1119G was also shown to have a bactericidal activity of >50% in 10 of the 15 isolates studied and could increase C3 deposition on the remaining five strains which resisted direct killing. In a mouse vaginal colonization model, FHD1119G reduced the bacterial load over the course of the infection and the median time to clearance from 7 to 5 days.

The second anti-virulence strategy targeting gonococcal LOS sialylation makes use of analogues of CMP-NANA, such as Leg5,7Ac_2_ and Neu5Ac9N_3_ (collectively termed CMP-nonulosonates or CMP-NulOs). When grown in the presence of CMP-NulOs, these analogues were successfully incorporated into gonococcal LOS by Lst without conferring resistance to complement mediated killing [116]. Further investigation revealed that Leg5,7Ac_2_ reduced factor H binding to levels equivalent to unsialylated gonococci, reduced clearance time of gonococcal infections in mice, and was able to block serum resistance even when added to the medium following the addition of CMP-NANA [118]. Leg5,7Ac_2_ was also shown to not be incorporated onto the surface of human B lymphoma cells, indicating that it may potentially be safe for use in humans [118]. Interestingly, the main mechanism by which CMP-NulOs provide protection in mouse models is by protecting against cathelicidins, not by inducing resistance to complement [119].

Several obstacles to the use of anti-sialic acid-based therapeutics exist. Sialidases expressed by the microbial flora of the vagina may de-sialylate gonococcal LOS, rendering FH/Fc based approaches ineffective [219]. Differences in the interaction of gonococci with the male and female genital tracts may also affect the efficacy of potential therapies [63].

#### 6.1.3. Lysozyme Inhibitors

The location of gonococcal colonization (e.g., urethra, pharynx, rectum, cervix, and conjunctiva) is rich in lysozyme, produced as part of the innate immune system or in macrophages, neutrophils, and dendritic cells [220,221,222,223]. Lysozyme is an antimicrobial protein that causes cell lysis and death through glycosidic bond hydrolysis between the carbohydrate motifs that make up the PG layer [224,225].

*N. gonorrhoeae* encodes two direct lysozyme inhibitors, surface-exposed lysozyme inhibitor of c-type lysozyme (SliC) and *N. gonorrhoeae*-adhesin complex protein (*Ng*ACP) [121,122]. The expression of these inhibitors is upregulated and essential for survival when exposed to lysozyme. In the study by Ragland et al. (2018) [121], mutants lacking either SliC, *Ng*ACP or both were constructed and tested against lysozyme from a variety of sources (i.e., human lysozyme, pooled human tears or pooled human saliva, and neutrophils). The loss of *Ng*ACP resulted in a significantly reduced gonococcal survival when exposed to lysozyme or neutrophils which SliC alone could not compensate for. However, these in vitro experiments did highlight the importance of both inhibitors in lysozyme resistance as the double mutant exhibited an increased sensitivity to lysozyme over either single mutant. SliC was found to play an important role in vivo survival as shown through experimental infection of female mouse genital tract. Mice infected with a strain lacking SliC resulted in a 3-, 372-, and 198-fold lower recovery than the wild-type strain on days 1, 3, and 5 post-inoculation. The same experiment in lysozyme defective mice supported the importance of SliC during in vivo infection.

Both SliC and *Ng*ACP are attractive targets for anti-virulence therapy and as potential vaccine candidates due to their extracellular localization, expression during human infection, and relative conservation among gonococcal strains [123,226,227]. No studies have yet to be published regarding the development of inhibitors or vaccine trials using SliC and *Ng*ACP but the structure of *Ng*ACP has been solved [123] and can be used to pursue structure-based drug design.

#### 6.1.4. PG O-Acetyltransferase B

Similar to the lysozyme inhibitors *Ng*ACP and SliC, the enzyme PG *O*-acetyltransferase B (PatB) provides protection against lysozyme-induced lysis. In addition, it plays a role in regulating gonococcal cell autolysis by preventing PG degradation. PatB is hypothesized to function together with PatA (an integral IM protein) as a two component system, whereby PatA translocates the presumed substrate acetyl-CoA to PatB in the periplasm, which then acts as a substrate for acetyl group addition onto the C-6 hydroxyl group of *N-*acetylmuramic acid [124,125,127,128,133,228]. Preventing *O*-acetylation of PG is key in mitigating the detrimental downstream effects of large circulating gonococcal *O*-acetylated PG fragments, such as arthritis and PG-mediated complement consumption [126,130,229,230] whilst returning sensitivity to lysozymes present in the host immune system. In addition, compounds targeting this enzyme will have the added benefit of not affecting the existing microbiota in the host that do not acetylate their PG.

In the study by Brott et al. (2019) [129], inhibitors were identified using high throughput screening that monitored hydrolysis of a fluorescent substrate, 4-methylumbelliferyl-acetate. Following validation pilot screens, optimized screening conditions and stringent statistical parameters were used to eliminate false positives. The remaining 12 compounds were put through dose response assays, followed by fluorescence quenching assays that removed potential hits with chemical properties that interfered with the assay. The compound 89224 was identified as a mixed/non-competitive inhibitor of *patB* with a K_i_ of 126 ± 19.5 µM. This compound is a benzothiazolyl-pyrazolo-pyridine derivative specific for *O*-acetylated PG, as evidenced by bacteriostatic growth inhibition of *N. gonorrhoeae* but not *E. coli*.

#### 6.1.5. Lytic Transglycosylase A

Lytic transglycosylases, in particular lytic transglycosylase A (LtgA) and LtgD, are involved in PG turnover through the cleavage of the glycosidic bond between *N*-acetylmuramic acid and *N*-acetylglucosamine, which results in the formation 1,6-anhydromuramic acid-based structures [75,132]. LtgA and LtgD are OM proteins, localizing in the cell septum and in discrete focal points around the bacterium, respectively [134]. Loss of LtgA and LtgD results in markedly reduced PG monomer release and increased sensitivity to killing by neutrophils that is independent from monomer release [135]. In *N. meningitidis*, an LtgA active site mutant strain had a detrimental effect on bacterial cell growth, division, and separation. In an in vivo mouse model, the mutant strain was cleared quicker and had a reduced cytokine production level [137].

The compound bulgecin A was found to bind specifically to a soluble lytic transglycosylase in *E. coli* and has been shown to have a synergistic effect when used with β-lactams to cause bulges in the cell wall of a variety of Gram-negative species [231,232,233,234,235,236]. Williams et al. (2017) investigated the effects of bulgecin A in pathogenic *Neisseria* in addition to solving the structure of LtgA from *N. meningitidis* complexed with bulgecin A [136]. The solved complex showed bulgecin A occupying the conserved active site of LtgA, suggesting that it acts as a competitive inhibitor and demonstrated the effect of bulgecin A on the ability of LtgA to facilitate 1,6-anhydro-muropeptide release using in vitro inhibition experiments. This study also demonstrated the synergistic effect of bulgecin A with β-lactams as seen in the lowered MIC values for penicillin G, amoxicillin and cefotaxime against pathogenic *Neisseria*.

### 6.2. Anaerobic Survival

Evidence of biofilm formation in cervical infections supports the persistence of gonococcal disease in women as the matrix protects against antibiotics and host defenses [237,238]. Due to this matrix, there exists a concentration gradient of oxygen and nutrients, suggesting that the bacteria can grow under anaerobic conditions. Several different genes are upregulated in response to anaerobic growth, including *aniA* (nitrite reductase) [239] and *norB* (nitric oxide reductase) [240].

Anaerobically induced protein A (AniA) is the only anaerobically induced OM protein that is undetected during aerobic growth [241] and reduces nitrite to nitric oxide. The presence of antibodies to AniA in the sera of patients diagnosed with gonorrhea or PID strongly suggests that AniA is expressed during pathogenesis [242]. Since AniA is present in all strains of *N. gonorrhoeae* and is essential for the growth and survival of *N. gonorrhoeae* under anaerobic conditions and for biofilm formation, it has become a target for both vaccine and inhibitor studies [23,139,140,141].

The inhibitor study by Sikora et al. (2017) [141] used a phage display approach to identify ligands interacting with AniA. From a large initial library of peptides, 29 peptides were identified and further examined using an enzyme-linked immunosorbent assay. The results of this assay and computational docking studies revealed that the inhibitor C7-3 was the most promising, binding near the type 2 copper site of the enzyme responsible for interaction with nitrite. Subsequent experiments with C7-3 and its derivatives, C7-3m1 and C7-3m2, demonstrated potent inhibition of AniA and antimicrobial activity against anaerobically grown *N. gonorrhoeae* strain 1291, which has resulted in potential commercialization of these materials [142].

### 6.3. Efflux Pumps

Most drug efflux proteins belong to five distinct families: the resistance-nodulation-cell division (RND), major facilitator, staphylococcal/small MDR, ATP-binding cassette, and multidrug and toxic compound extrusion families [243]. In gonococci, four efflux pump systems, MtrCDE, MacAB, NorM, and FarAB, have been identified in all strains [244,245,246,247]. The MtrCDE system belongs to the RND family and has been shown to recognize antimicrobials previously or currently recommended for gonorrhea treatment [46,248,249,250].

A major gonococcal AMR determinant is the MtrCDE pump. It is composed of IM and OM channels (MtrD and MtrE, respectively), which are connected through a periplasmic membrane fusion lipoprotein (MtrC) [244,251,252]. Expression of *mtrCDE* is directly regulated by the MtrR repressor and MtrA activator [253,254,255]. Mutations causing the overexpression of MtrCDE can occur in MtrR or in the promoter region of the *mtrCDE* operon, conferring increased resistance to antibiotics such as azithromycin [244,248]. Jerse et al. (2003) [99] found that mutations in *mtrCDE* reduced gonococcal survival in the female murine genital tract. Additionally, Chen et al. (2019) [147] showed that transcriptional repression of the MtrCDE efflux pump in penicillin resistant strains could increase the penicillin susceptibility to therapeutic levels in mice models. MtrCDE may also contribute to in vivo gonococcal survival by protecting against the antimicrobial effects of fatty acids and CAMPs found at mucosal surfaces [248,256].

Efflux pump inhibitors have been considered for the treatment of gonorrhea for quite some time as *mtrCDE* is expressed by gonococci in the human urogenital tract of both men and women [226,257]. However, the current candidates under development such as the efflux pump inhibitor MC-207110 (phenylalanine arginine β-naphthylamide) have been associated with high levels of host cell toxicity and unfavorable pharmacokinetic properties [143,145,258].

### 6.4. Protein Folding Pathways

The process of protein folding is crucial for ensuring that proper biological activity and conformational stability is achieved as protein misfolding in prokaryotic cells can lead to aggregation into insoluble inclusion bodies [259]. As such, bacteria contain several mechanisms that prevent misfolding from occurring. These molecular chaperones facilitate native protein stabilization, translocation, re-folding, and degradation, and include proteins such as heat-shock proteins [260,261], peptidyl-prolyl cis–trans isomerases (PPIases) [262] and oxidoreductases [152,263].

#### 6.4.1. Macrophage Infectivity Potentiator

Macrophage infectivity potentiator (Mip) proteins are members of the FK-506 binding protein subfamily, belonging to the immunophilin superfamily. This protein family exhibits PPIase activity, thereby catalyzing the cis–trans isomerization of peptide bonds directly preceding a proline residue [262]. This is an inherently slow reaction and can be rate limiting in the correct folding of various proteins in the absence of a PPIase protein [264,265].

The Mip protein in *N. gonorrhoeae* is an OM protein found to be present, with a high degree of similarity, in all 20 clinical strains tested by Starnino et al. (2010) [266]. In addition, all infected patients’ sera were able to recognize recombinant *Ng*Mip protein, indicating immunogenicity. This was reinforced by the work of Humbert and Christodoulides (2018) [267] which showed that recombinant *N. meningitidis* Mip can produce bactericidal antibodies that are effective against both *N. meningitidis* and *N. gonorrhoeae* strains. Further, a *N. gonorrhoeae* strain lacking *Ng*Mip showed decreased survival within murine RAW 264.7 macrophage cells [150]. The ability of a *N. meningitidis* strain lacking *Nm*Mip to grow in human whole blood was decreased in comparison to the parent control [268]. These data indicate the importance of the Mip protein in the virulence of pathogenic *Neisseria* species.

Novel inhibitors originally designed against the Mip protein of *Legionella pneumophila* and *Burkholderia pseudomallei* were tested against *N. gonorrhoeae* and *N. meningitidis* by Reimer et al. (2016) [151]. The cognate inhibitor of Mip proteins, rapamycin, was used as a basis for the synthesis of these pipecolic acid derivative inhibitors, and the high level of conservation of Mip proteins across bacterial species allowed for successful screening across multiple pathogens. The two inhibitors studied, PipN3 and PipN4, were able to inhibit the PPIase activity of recombinant gonococcal Mip, as well as reduce intracellular survival of *N. gonorrhoeae* in PMNs. Treatment with PipN3 and PipN4 also reduced the ability of *N. meningitidis* to adhere to and invade human nasopharyngeal Detroit 562 epithelial cells.

#### 6.4.2. Oxidative Protein Folding System

Disulfide bond protein A (DsbA) and DsbB are periplasmic oxidoreductases required for disulfide bond formation in protein substrates. DsbA is a periplasmic protein that belongs to the thioredoxin superfamily, with an active site CXXC motif embedded in a thioredoxin-like fold and a highly conserved *cis*-proline in an adjacent loop. DsbA catalyzes the formation of disulfide bonds between thiol groups of two cysteine residues [263]. DsbA is kept in an oxidized state by DsbB reductase, which transfers electrons to quinone through the electron transfer system [269]. DsbB is an inner membrane protein and a member of the vitamin K epoxide reductase superfamily [152]. *N. gonorrhoeae* encodes two DsbA oxidoreductases. DsbA1 is a lipoprotein bound to the inner membrane, while DsbA3 is a soluble periplasmic protein [154].

Inactivation of the DsbA/DsbB oxidative system has pleiotropic effects on various virulence-associated phenotypes and decreases survival in in vivo infection models of many Gram-negative pathogens [166]. Inactivation of *dsbA1/dsbA2* in *N. meningitidis* causes the inefficient folding of PilE and PilQ, resulting in reduced colonization and competence [154,156], while inactivation of *dsbA3* results in instability and loss of function in EptA [270]. At this stage, no studies have characterized the effects of *dsbB* loss in *Neisseria* species.

Previous studies have identified small-molecule inhibitors, phenylthiazole, benzofuran, and pyridazinone derivatives, against the DsbA/DsbB system in *E. coli*. Pyridazinone-based compounds [161,163,165,169] bound to *Ec*DsbB at the quinone-binding site between the first two transmembrane segments, competing with quinone, or to a segment of the second periplasmic loop that interacts with *Ec*DsbA [164,271]. Phenylthiazole and benzofuran-based compounds bound to the hydrophobic groove of *Ec*DsbA, which is required for interaction with *Ec*DsbB [163,169]. Phenylalanine and tyrosine-based phenylthiazole derivatives were also found to selectively inhibit *Ec*DsbA in in vitro assays, with reduced motility in soft agar and no effect on growth in liquid media [163]. However, these compounds have not been trialed in *N. gonorrhoeae*.

### 6.5. Adhesion and Invasion

As described earlier, pathogenic *Neisseria* species express numerous features that facilitate the attachment and invasion of host cells to begin the cycle of infection. The first step of infection relies heavily on attachment and colonization through microcolony formation on the epithelial cell surface [70,272]. This process is mediated by tfp, an OM structure that is also responsible for enabling transformation competence, immune evasion through antigenic and phase variation, twitching motility, and protection from CAMP-, ROS- and PMN-killing mechanisms [273,274,275,276,277,278,279,280,281]. Additionally, gonococci express Opa proteins that are important for facilitating attachment to host cells via glycan binding [65,66,187,282]. Therefore, tfp and Opa proteins represent attractive targets that prevent gonococcal-host interactions.

#### 6.5.1. Type IV Pili

Tfp are long filamentous structures extending from the inner membrane to the bacterial surface, passing through the outer membrane via PilQ [283,284]. It is composed of the major pilin, PilE, and other minor pilins such as ComP, PilV, PilC and PilH-L [173,177,272,281,285,286]. The tfp is a highly dynamic structure which undergoes rapid cycles of extension and retraction mediated by PilF and PilT, respectively [175,176,287].

Two recent studies have identified inhibitors of neisserial tfp. The first inhibitor, referred to as compound B in the publication, was identified through a phenotypic screen and successfully prevented the adherence and formation of *N. meningitidis* microcolonies on the human umbilical vein endothelial cell surface [181]. Cellular and in vitro experiments showed that compound B could inhibit the PilF ATPase enzymatic activity resulting in lowered surface expression levels of tfp. Since compound B did not show any inhibitory activity on PilT, this strongly indicates an inhibitory effect on tfp assembly. Additionally, compound B could also prevent the autoaggregation of *N. gonorrhoeae* and induced the disaggregation of preformed gonococcal aggregates, indicating a potential broad-spectrum application. In the same study, structure–activity relationship analysis of compound B showed that the 2,4-dimethoxybenzoyl and piperidine moieties can be modified without affecting efficiency. As such, these components could be modified in future studies to obtain more soluble and stable inhibitors.

The second inhibitor trifluoperazine and related phenothiazines are part of a group of anti-psychotic drugs. Unlike compound B, the inhibitors identified by Denis et al. (2019) [182] do not directly target tfp. Instead, the phenothiazine derivatives affect the function of the Na^+^-pumping NADH:quinone oxidoreductase (Na^+^-NQR) in *N. meningitidis* and *N. gonorrhoeae*, which result in a reduction in tfp twitching motility and the dispersal of bacterial aggregates [182]. Mice infected with *N. meningitidis* treated with the both phenothiazine and antibiotics had reduced bacteremia and increased survival, highlighting the importance of preventing tfp-mediated pathogenesis.

The effects of both compounds on piliation were fast acting, reflecting the rapid dynamics of the tfp [181,182]. Additionally, both studies showed that although these compounds were initially designed to inhibit *N. meningitidis*, they are also effective on other Gram-negative tfp-expressing bacterial pathogens such as *N. gonorrhoeae* and *P. aeruginosa*. Further investigations into targeting tfp should be performed due to the broad range of pathogens that rely on piliation as a virulence factor.

A separate approach to blocking tfp-mediated attachment to host cells has been recently described by Poole et al. (2020) [183] who screened a library of FDA-approved drugs for binding to the I-domain of complement receptor 3 (CR3). They retrieved two drugs, methyldopa and carbamazepine, which bound with high affinity to the CR3 receptor. Using a docking model, they also synthesized a peptide, G2, which bound with such high affinity to the I-domain of CR3 that it inhibits tfp-mediated gonococcal colonization of primary cervical cells.

#### 6.5.2. Mannose-Binding (Opa) Proteins

Opa proteins are OM proteins that promote intimate adhesion to CEACAM and glycans on host epithelial cells, and are observed to be expressed by gonococci isolated from human male models of infection [184,185,188]. Cole et al. (2010) [288] showed that Opa proteins promote persistent late stage of infection in the female murine genital tract. This study and another by Koch (1947) [289] suggested that expression of Opa variants may have a link to stages of the menstrual cycle.

A study by Semchenko et al. (2019) [190] used a glycan array analysis to investigate the glycan binding profile of *N. gonorrhoeae* and the proteins that mediate this interaction. The highest percentage of bound glycans were glycosaminoglycans, such as HSPG, and mannosylated glycans. Using surface plasmon resonance experiments, the glycan that had the highest-affinity interaction was α1-2-mannobiose and liquid chromatography-mass spectrometry was used to successfully identify three Opa proteins that were the most abundant mannose-binding proteins. A second surface plasmon resonance assay confirmed that Opa-expressing gonococci had a 6- to 27-fold higher affinity to mannosyl glycans than Opa-nonexpressing gonococci. Since mannose was found on genital tract epithelial cells, Semchenko and collaborators performed infection inhibition assays using the mannose-binding lectin ConA or α-methyl D-mannoside (mannose-binding protein antagonist) pretreated epithelial cells. Using either inhibitor resulted in a clear reduction in gonococcal adherence to primary cervical epithelial cells and urethral epithelial cells. These results affirmed the need for the development of inhibitors specific to Opa/mannose-binding proteins as gonococcal anti-infectives.

## 7. Considerations for Further Clinical Development of AVTs

Although there is a considerable number of AVTs for many bacterial pathogens in pre-clinical development [106,290], licensing pathways for these compounds remain largely underdeveloped. Provisionally, if the compounds are novel, they are likely to progress via the same regulatory pathway as antibiotics, which may take as long as 10–15 years (Table 2) [291]. However, a shortened licensing pipeline of 3–12 years is possible if the AVT is discovered in previously FDA-approved compound library and shows efficacy in phase 2 trials [292].

One outstanding advantage for the development of AVTs against *N. gonorrhoeae* is that this field has access to male human models of infection in the pre-clinical development phase [179,293]. Human models of infection can be used to validate the chosen target for AVT development and establish the end points (e.g., no colonization by the pathogen or reduction in symptoms), which can be then used to develop dosing strategies for phase 1 trials. Three AVT targets—EptA, tfp and Opa mutants—have been tested in human models to validate them for therapeutic intervention (Table 1). Phase 0 trials of fewer than 15 people could be used to examine the efficacy of AVTs and could be used as go-no-go breakpoints for candidates that will progress to the more expensive and lengthy phase 1 and 2 trials [292]. This should reduce the failure rates in the development of AVTs and shorten their time to licensing (Table 2).

Dependent upon the mode of action of an AVT, some may have properties more similar to antibiotics while others act as adjuvants to antibiotic therapy [295]. Vaccines are targeted against pathogens with generally no or very little cross-over against other microbial species and are administered pre-emptively against infection, while antibiotics are administered to cure acute symptomatic infections. In the case of *N. gonorrhoeae*, symptomatic infections in men and PID in women are treated with antibiotics at a late stage of the infection where inflammation may cause long-term side effects such as infertility [296] and adverse outcomes for pregnancy [297]. Thus, although antibiotic treatment suppresses further transmission in the community, most successfully via males, intervention is too late to either completely resolve transmission in the community or prevent long-term morbidity from asymptomatic infections in women. AVTs that could be applied preemptively in the community to suppress asymptomatic transmission are likely to have the highest benefit, particularly for women who are at the highest risk of developing PID which increases the risk of infertility. Modes of delivery that would most likely benefit women would either involve oral delivery or direct topical applications via hygiene products such as commercial vaginal microbicides, some of which have viricidal and anti-chlamydial properties [298,299,300,301].

## 8. Conclusions

Given the challenges in the development of antibiotics and vaccines against gonorrhea, AVTs are a viable alternative, especially where candidate targets have been validated in human models of infection and correlates for protection have been established. AVTs may find roles as antibiotic adjuvants [295] for traditional antibiotic therapy to reverse development of antibiotic resistance or may find a wider role as an intervention that can reduce asymptomatic infections which drive outbreaks and increase the risk of long-term morbidity in women.

## Figures and Tables

**Table 1:** Summary of anti-virulence targets in *N. gonorrhoeae*.

Anti-Virulence Target	Function	Target Validation			Available Inhibitors	Inhibitor Studies	References
		In Vitro Testing	Structural Studies	In Vivo Models			
**Bacterial cell wall maintenance and modification**							
EptA	Catalyzes the addition of pEtN onto lipid A of the OM.	Loss of EptA increases susceptibility to killing by PMNs, macrophages, CAMPs and human serum.	Full structure solved of *N. meningitidis* homologue (98% identity, PDB accession code 5FGN).	Reduced survival rates of *eptA* mutant in mouse and human models.	N/A	N/A	[83,108,109,110,111,112]
							
Lst	Catalyzes the addition of *N*-acetyl-neuraminic acid onto lacto-*N*-neotetraose of LOS. Primary mechanism for resistance to human complement.	Loss of Lst increases susceptibility to killing by PMNs and human serum.	Full structure of *N. meningitidis* homologue apo form (92% identical, PDB accession code 2YK4 ^1^) and with structural donor sugar analogs or products solved (PDB accession code 2YK5, 2YK6, and 2YK7 ^1^).	Reduced survival rates of *lst* mutant in mouse models.	FHD1119G and Leg5,7Ac_2_	Increased serum sensitivity. Significantly reduced duration and burden of infection in mouse vaginal colonization model.	[113,114,115,116,117,118,119]
							
*Ng*ACP and SliC	Essential for survival against lysozyme.	Loss of *Ng*ACP and SliC increased susceptibility to human lysozyme. *Ng*ACP loss significantly reduced survival in PMNs.	Mature *Ng*ACP structure has been solved (PDB accession code 6GQ4). Structure of SliC homologue in *Pseudomonas aeruginosa* (MliC) solved (23.3% identity, PDB accession code 3F6Z ^1^).	Reduced survival rates of *sliC* mutant in mouse models.	N/A	N/A	[120,121,122,123]
							
PatB	Catalyzes *O*-acetylation of *N*-acetyl-muramic acid.	Increased sensitivity to lysozyme in human sera or lysozyme purified from human PMNs.	Structure of PatB homologue in *Staphylococcus aureus* (OatA C-terminal catalytic domain) has been solved (15% identical, PDB accession code 6VJP ^1^).	N/A	Compound 89224	Treatment reduced bacterial growth by 90%. Inhibitor binding studied using microtiter plate-based fluorometric assay.	[124,125,126,127,128,129,130,131]
							
LtgA	Catalyzes cleavage of *N*-acetyl-muramic acid-β-1,4-*N*-acetylglucosamine to form PG monomer fragments during cell growth.	Reduction in PG monomer release. Loss of LtgA in *N. meningitidis* has a detrimental effect on bacterial cell growth, division, and separation.	Structure of *N. meningitidis* homologue (97% identical, PDB accession code 6FPN ^1^).	*Nm*LtgA mutant cleared quicker than wild-type and reduced cytokine induction in mouse model.	Bulgecin A	Inhibited LgtA activity and had a synergistic effect with β-lactams.	[75,132,133,134,135,136,137]
**Anaerobic survival**							
AniA	Reduces nitrite to nitric oxide. Essential for anaerobic growth.	Loss of AniA reduces anaerobic growth and biofilm formation.	Soluble domain structure solved (PDB accession code 1KBW, 1KBV, 5TB7, and 5UE6).	Immunization with a truncated form of AniA generates protective antisera in a mouse model.	C7-3	Significantly inhibited enzyme activity and gonococcal growth under anaerobic conditions. Inhibitor binding studied using molecular docking and biolayer interferometry. A patent has been approved for C7-3 and its derivatives.	[138,139,140,141,142]
**Efflux pump**							
MtrCDE	Selective efflux of antimicrobials resulting in increased resistance to penicillins, macrolides and extended spectrum cephalosporins.	Loss of the MtrCDE pump results in increased susceptibility to penicillin, ceftriaxone, azithromycin, tetracycline, and solithromycin in the WHO clinical panel of multidrug-resistant (MDR) strains.	Full structures of MtrD and MtrE solved (PDB accession code 4MT1 (MtrD), 4MT0 (MtrE), 6VKS (MtrD from strain CR103 in complex with ampicillin) and 6VKT (MtrD from strain CR103 in complex with erythromycin). Full structure of MtrC homologue (MexA) in *P. aeruginosa* solved (43% identity, PDB accession code 1VF7 ^1^).	Loss of the MtrCDE pump reduced gonococcal survival and increased penicillin susceptibility to therapeutic levels in mouse models.	Phenylalanine arginine β-naphthylamide (PaβN)	Untested in *N. gonorrhoeae*, derivatives have been halted due to high host cell toxicity.	[46,99,143,144,145,146,147,148]
**Protein folding pathways**							
Mip	Catalyzes the *cis–trans* isomerization of peptide bonds directly preceding a proline residue.	Loss of Mip decreased gonococcal survival within macrophages and PMNs.	Full structure of *Legionella pneumophila* homologue solved (44.8% identical, PDB accession code 1FD9 ^1^).	N/A	PipN3 and PipN4	Compounds inhibited enzyme activity and reduce gonococcal survival in neutrophils.	[149,150,151]
							
DsbA/DsbB	Catalyzes formation of disulfide bonds in OM proteins involved in virulence.	Loss of DsbA1 in *N. meningitidis* affects tfp function, reducing colonization and competence.	Full structures of *N. meningitidis* homologues DsbA1 (97% identical, PDB accession code 3DVW and 3A3T ^1^) and DsbA3 (93% identical, PDB accession code 3DVX and 2ZNM^1^) solved. Full structure of *E. coli* DsbA/B complex homologue solved (28.7% identical, PDB accession code 3E9J ^1^).	N/A	Phenylthiazole, benzofuran, phenylthiophene or phenoxyphenyl derivatives	Untested in *N. gonorrhoeae*.	[152,153,154,155,156,157,158,159,160,161,162,163,164,165,166,167,168,169]
**Adhesion and invasion**							
Type IV pili	Essential for adhesion/colonization, horizontal gene transfer, twitching motility.	Tfp mutants lacking PilE are unable to adhere to human epithelial cells, are non-motile and are incompetent.	Tfp structure has been solved (PDB accession code 5VXX, 1AY2, 2HIL and 2HI2).	In the human male model of infection, men inoculated with a gonococcal *pilE* mutant developed watery urethral discharge or were asymptomatic.	Compound B	Prevents pilus elongation.	[170,171,172,173,174,175,176,177,178,179,180,181,182,183]
					Phenothiazines	Inhibited Na^+^-pumping NADH:quinone oxidoreductase. Tests with *N. meningitidis* reduced bacteremia and increased survival in a mouse model.	
					Compound G2, carbamazepine and methyldopa	Inhibits tfp binding to host receptor CR3 on primary cell line. Carbamazepine and methyldopa are re-purposed drug (FDA-approved anti-convulsant and high blood pressure medication, respectively).	
							
Mannose-binding (Opa) proteins	Required for adherence to host epithelial cells.	Opa-less bacteria do not adhere to Chinese hamster ovary cells.	Structure of Opa60 has been solved (76% identity, PDB accession code 2MAF ^1^).	Gonococci recovered from human models are always Opa positive, even if the inoculum was Opa negative.	ConA and α-methyl D-mannoside (Mannosides)	Compounds reduced gonococcal adherence to primary cervical epithelial cells and urethral epithelial cells.	[184,185,186,187,188,189,190]

^1^ PDB identification and percentage identity obtained using the NCBI BLASTp query of the PDB database [191,192,193].

**Table 2:** Characteristics of AVTs, antibiotics and vaccines *.

Characteristics	AVTs	Antibiotics	Hypothetical Vaccine
Mode of action	Selective inhibition of pathogens, preserves the microbiome	Broad spectrum killing of microorganisms, removes the microbiome	Selective inhibition of pathogen, preserves the microbiome
Mechanism of action	Tailored to prevent colonization, transmission, and infection by a pathogen	Kills systemic microorganisms—resolves acute infections. Not used for asymptomatic infections	Prevents acute infection by a pathogen. In some instances, vaccines can prevent colonization and transmission of the pathogen.
Use	Pre-exposure prophylaxis or therapeutic	Therapeutic	Pre-exposure therapeutic
Dose	Multiple dosing as needed	Multiple dosing, 3–4 days	1–3 doses
Route of administration	Oral, topical 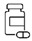 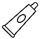	Oral, injectable 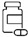 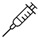	Injectable 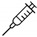
Correlate of protection	Absence of viable pathogen at the site of infection	Absence of viable pathogen at the site of infection	Currently unknown
Implementation	Pharmacy or medical prescription	Medical prescription	Primary care clinics
Size and cost of clinical trials	>1000 subjects ** <$1 billion	>10,000 subjects >$1.5 billion	>100,000 subjects >$1.8 billion
Drug development timeframe from pre-clinical to licensing	5–10 years **	9–15 years	9–15 years
Licensing pipeline	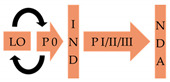	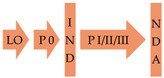	

* Information tabulated from Paul et al. (2010) [294] and Farha and Brown (2019) [291]. ** Provisional estimates as the licensing pipeline has not been fully established for AVTs against *N. gonorrhoeae*. LO: Lead optimization. P 0: Phase 0. P I/II/III: Phase I/II/III. IND: investigational new drug application. NDA: new drug application. FDA: United States Food and Drug Administration (FDA) review and approval.

## Data Availability

Not applicable.
